# Supplementary material for: Bacterial peptidoglycan acts as a digestive signal mediating host adaptation to diverse food resources in C. elegans
Source: Nat Commun. 2024 Apr 16;15:3286. doi: 10.1038/s41467-024-47530-y (PMC11021419; doi:10.1038/s41467-024-47530-y)
Supplement: Supplementary file 1 — Supplementary Information [file 41467_2024_47530_MOESM1_ESM.pdf]

Supplementary information

Bacterial peptidoglycan acts as a digestive signal mediating host adaptation to diverse food resources in *C. elegans*

Fanrui Hao, Huimin Liu, Bin Qi

Supplementary Figures:

Supplementary Fig. 1-Fig.7

Supplementary Fig. 1

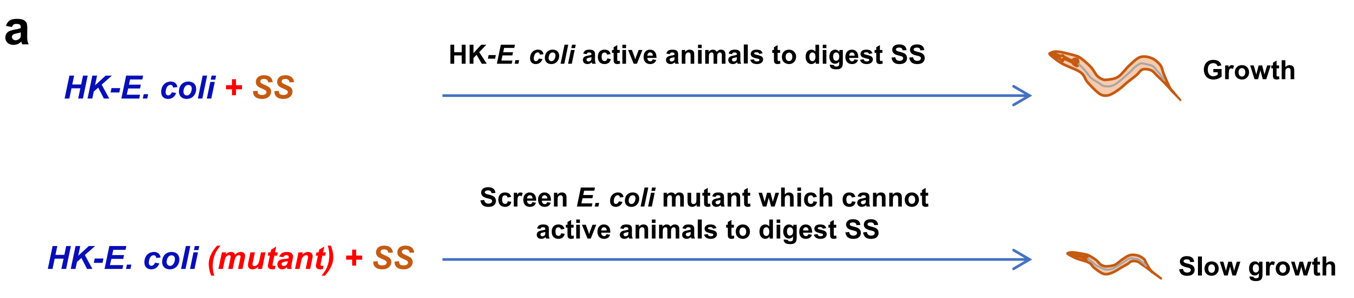

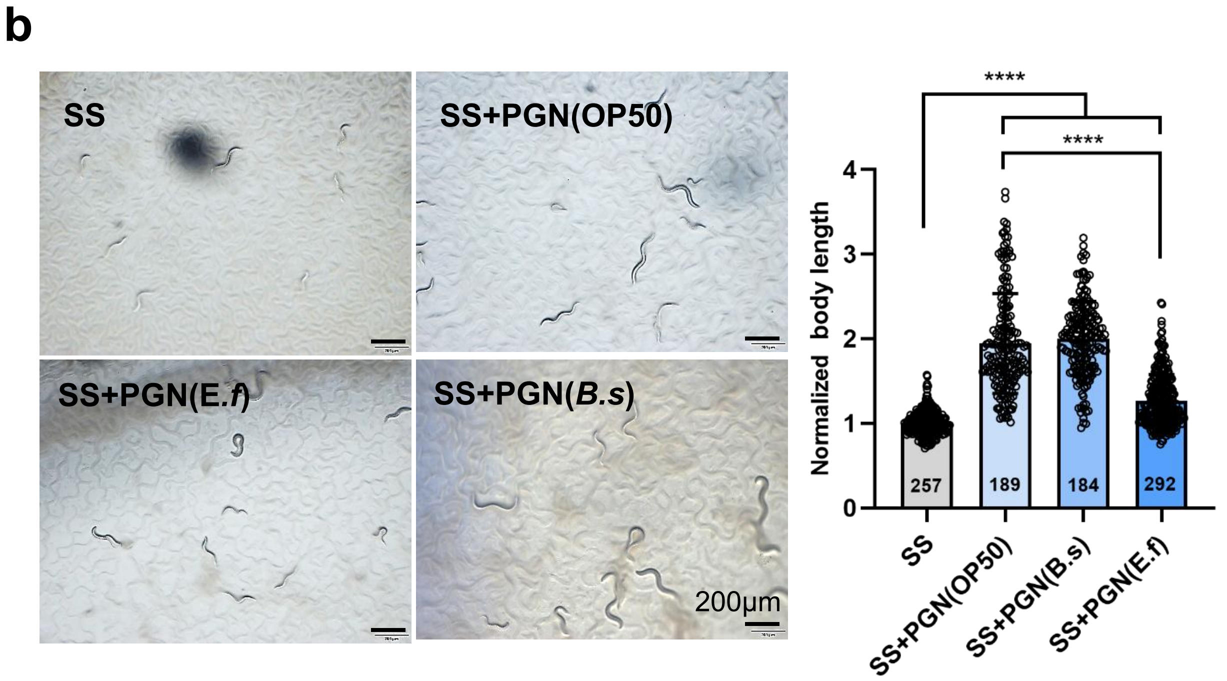

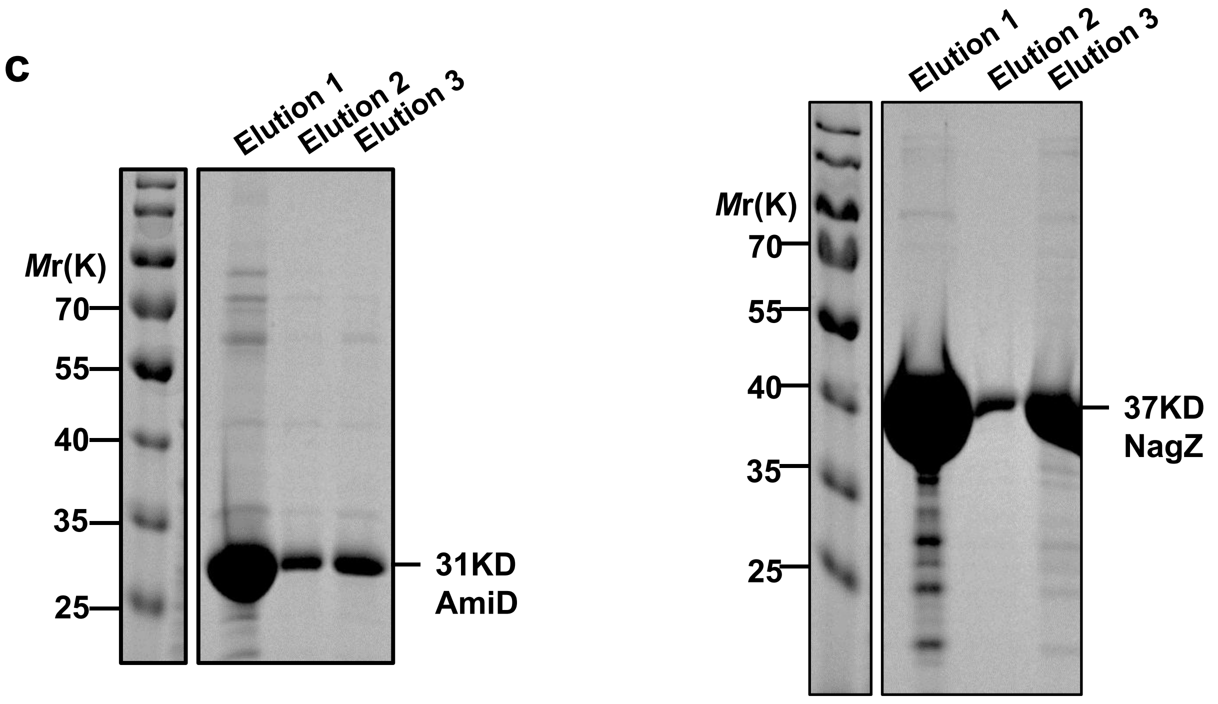

Supplementary Fig. 1: PGN activates animals to digest inedible food. Related to Fig. 1.

a Illustrated diagram depicting the screening strategy used to identify signals from HK-*E. coli* that activate animals to digest SS. Synchronized L1 WT animals were raised on HK-*E. coli* (mutant) + SS for 5 days to observe developmental phenotype. *E. coli* mutants lacking the digestive signal would be unable to stimulate animal digestion of SS to facilitate growth. **b** Developmental progression of animals grown on SS+PGN extracted from *E. coli, E. f* and *B.s* at 4d at 20℃. n=257 for SS, n=189 for SS+PGN(OP50), n=184 for SS+PGN(B.s), n=292 for SS+PGN(E.f). Statistical significance was calculated using multiple unpaired t-tests (two-tailed). Obtained *p* values were as follows: SS vs. SS+PGN(OP50), SS+PGN(B.s) and SS+PGN(E.f); ****$p<0.0001$, respectively. Scale bar, 200μm. **c** SDS-PAGE gel showing purified recombinant proteins (AmiD and NagZ). For all panels, n= number of animals which were scored from at least three independent experiments. Data are represented as mean ± SD. ****$p<0.0001$. Source data are provided as a Source Data file.

Supplementary Fig. 2

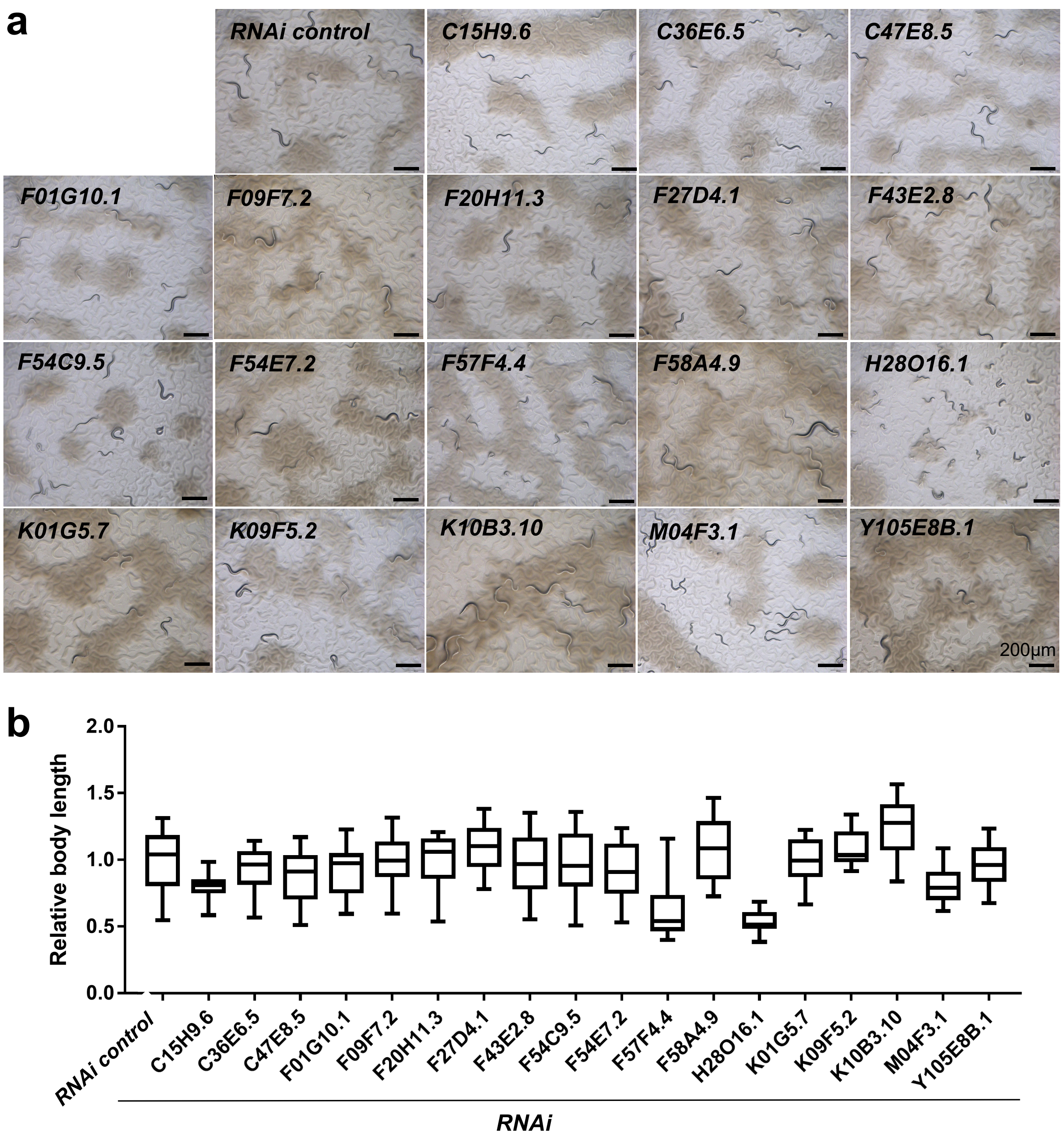

Supplementary Fig. 2: RNAi screen of gut specific expression genes which may be involved in digestion. Related to Fig. 2.
a, b Developmental phenotype (**a**) and quantification of body length (**b**) of animals with indicated RNAi grown on HK-*E. coli*+SS for 4 days at 20℃. Source data are provided as a Source Data file.

Supplementary Fig. 3

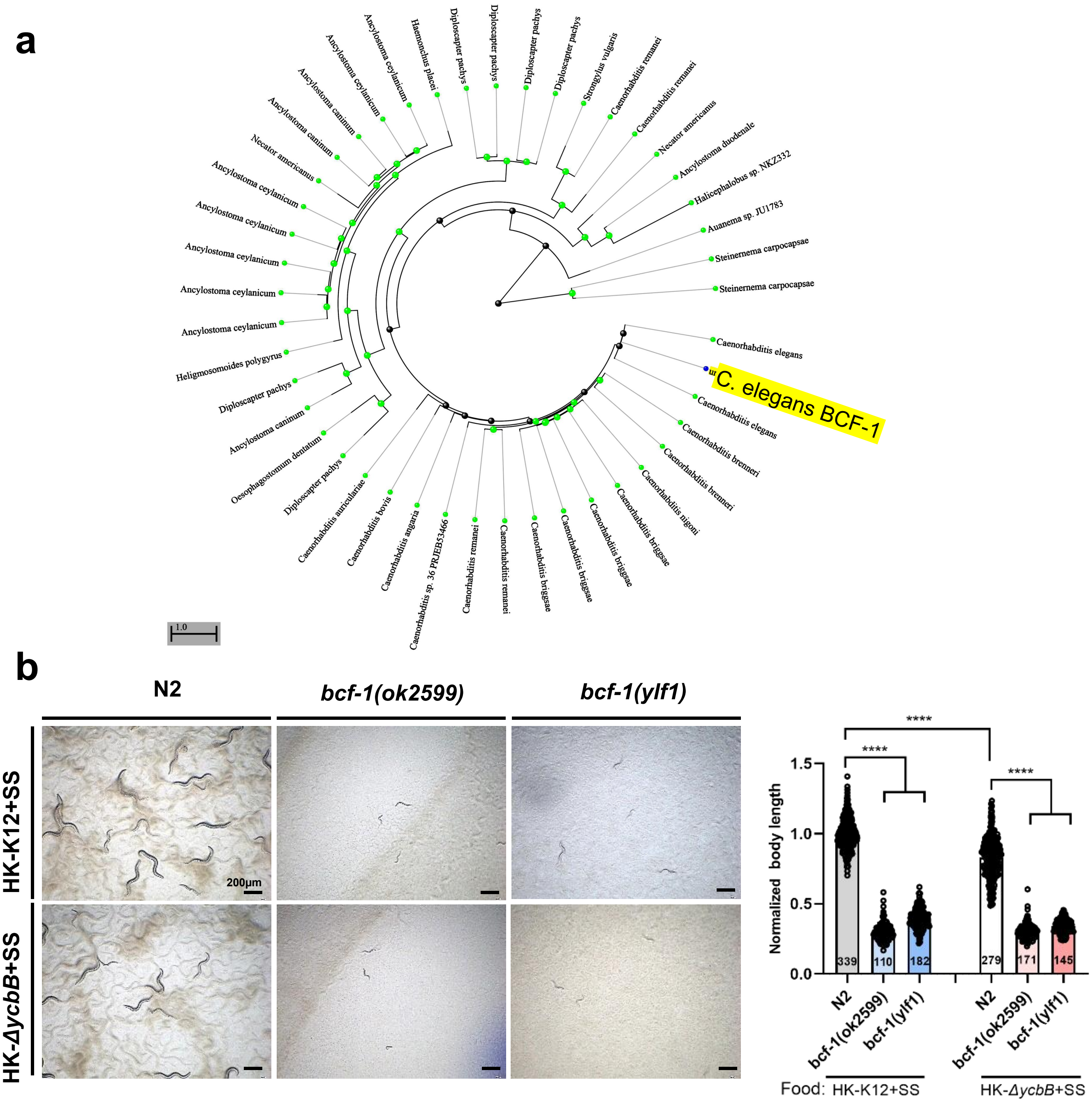

Supplementary Fig. 3: Phylogenetic tree of BCF-1 in nematodes. Related to Fig. 3.

a Phylogenetic tree showing that *C. elegans* BCF-1 protein is conserved in nematodes, especially in *C. remanei, C. nigoni, C. briggsae, C. bovis, C. auriculariae*, *and C. angaria.* **b** Development phenotype of N2 and *bcf*-1 mutants grown on the HK-*E. coli* (*ΔycbB* mutant) + SS or SS at 20℃ for 3d post-L1 synchronization. n=339, 110 and 182 for N2, *bcf-1(ok2599)* and *bcf-1(ylf1)* on HK-K12+SS.n=279, 171, 145 for N2, *bcf-1(ok2599)* and *bcf-1(ylf1)* on HK-*ΔycbB*+SS. Statistical significance was calculated using multiple unpaired t-tests (two-tailed). Obtained *p* values were as follows:N2 vs. *bcf-1(ok2599)* and *bcf-1(ylf1)* on HK-*ΔycbB*+SS; ****$p<0.0001$. Scale bar, 200μm. Source data are provided as a Source Data file.

Supplementary Fig. 4

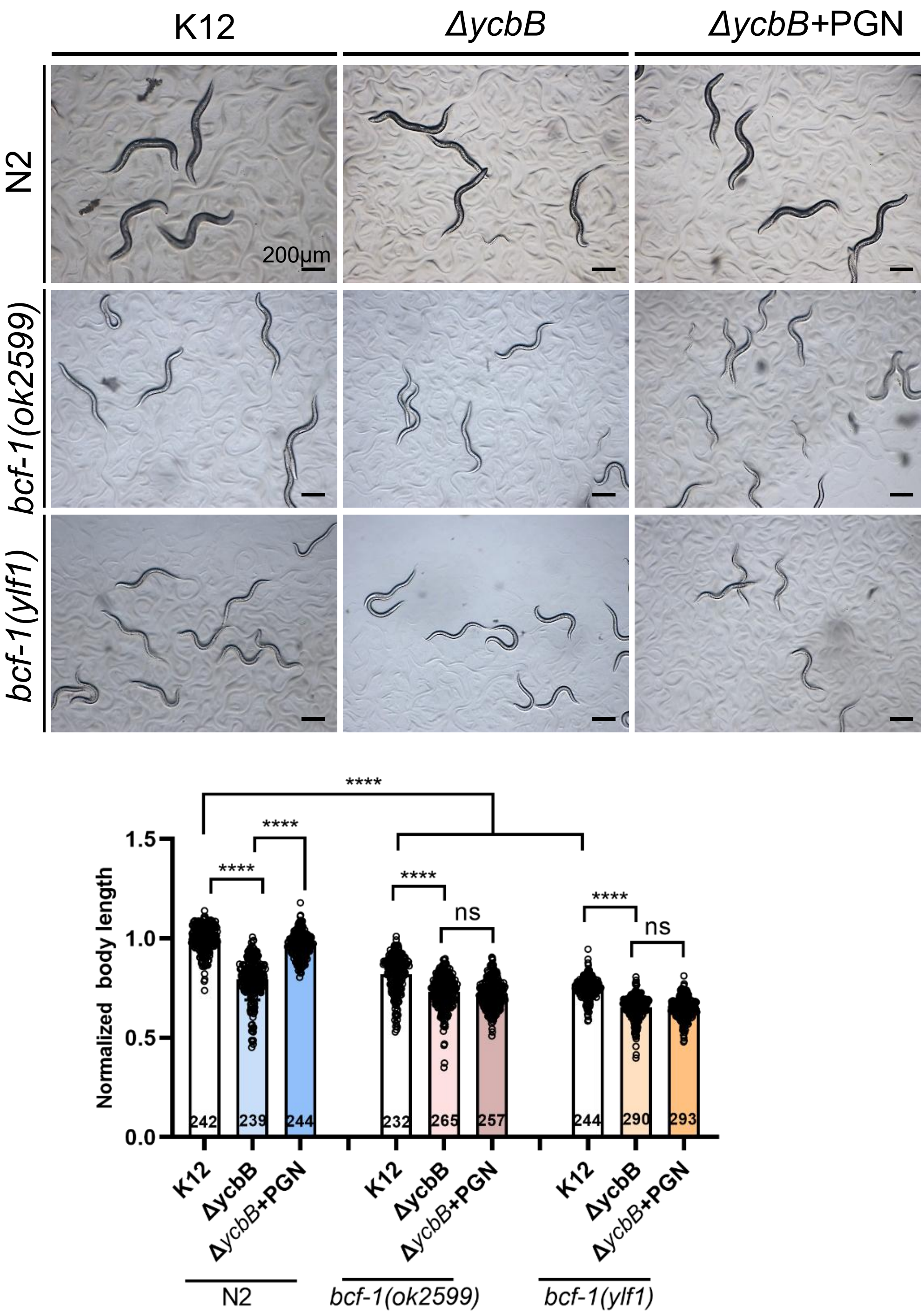

Supplementary Fig. 4: Development of added PGN in *ΔycbB*. Related to Fig. 4.

Development phenotype of N2 and *bcf*-1 mutants grown on the *ΔycbB* mutant or *ΔycbB*+PGN at 20℃ for 3d post-L1 synchronization. Scale bar, 200μm. n=242, 239 and 244 for N2; n=232, 265 and 257 for *bcf-1(ok2599)* and n=244, 290 and 293 for *bcf-1(ylf1)* on K12, *ΔycbB* and *ΔycbB*+PGN, respectively.

For all panels, n= number of animals which were scored from at least three independent experiments. Data are represented as mean ± SD. ****p<0.0001, ns: no significant difference. Source data are provided as a Source Data file.

Supplementary Fig. 5

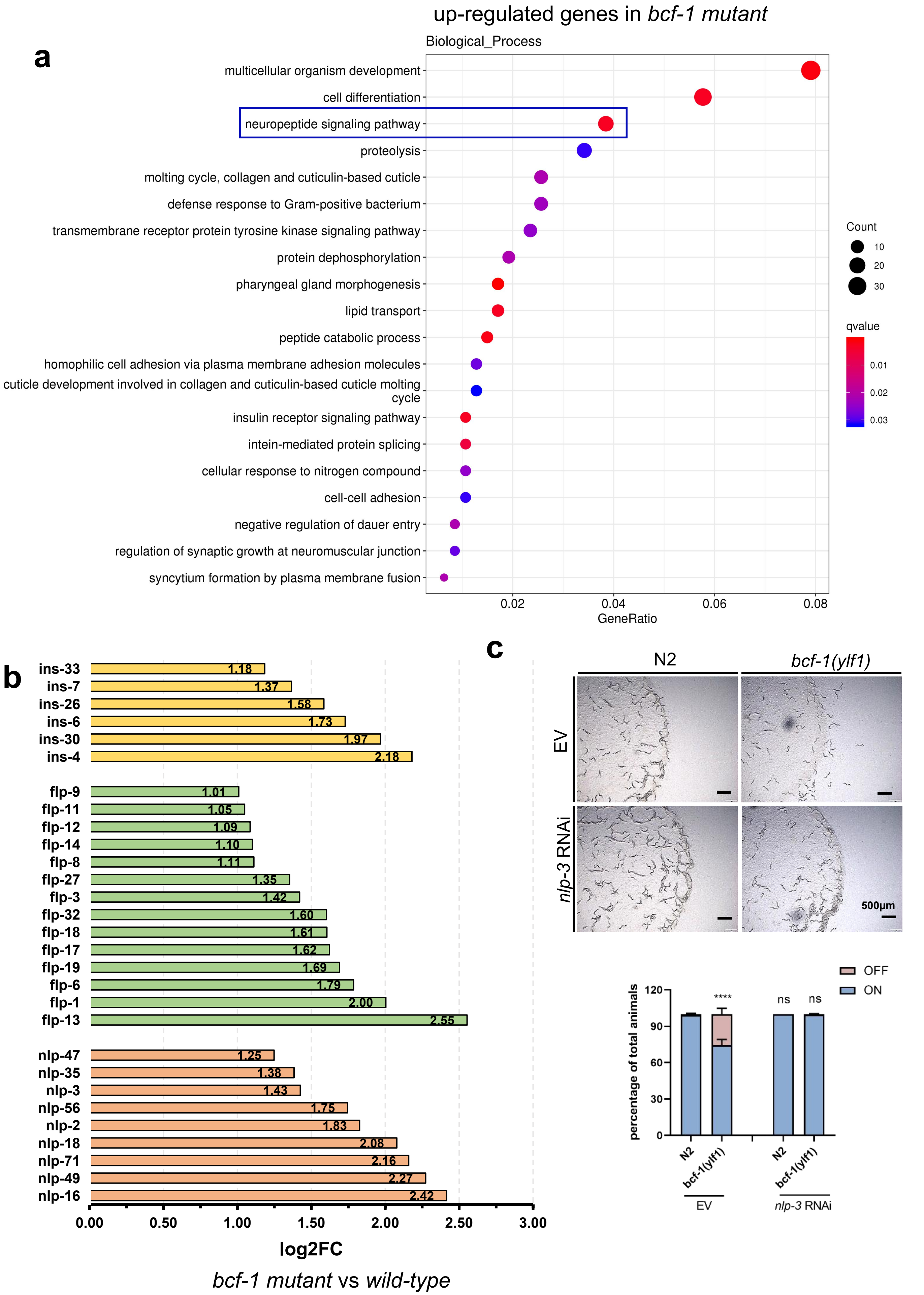

Supplementary Fig. 5: Upregulation of neuropeptide genes in *bcf-1* mutant. Related to Fig. 5.

a GO term enrichment analysis of upregulated genes in *bcf-1* mutant compared with wild-type N2 animals from RNA-seq data[1].

b RNA-seq data[1] shows upregulated neuropeptide genes in *bcf-1* mutant compared to N2.

c The food avoidance phenotype in *bcf-1* mutant after RNAi nlp-3. Food avoidance is rescued in animals with *bcf-1* mutation after RNAi *nlp-3*. 200–400 animals/assay, mean ± SD from 3 replicates. Obtained *p* values were as follows: N2 vs. *bcf-1(ylf1)* on EV; ****$p<0.0001$. N2 vs. *bcf-1(ylf1)* on *nlp-3* RNAi, $p>0.9999$, ns.

Data are represented as mean ± SD. ****p<0.0001, ns: no significant difference. Source data are provided as a Source Data file.

Supplementary Fig. 6

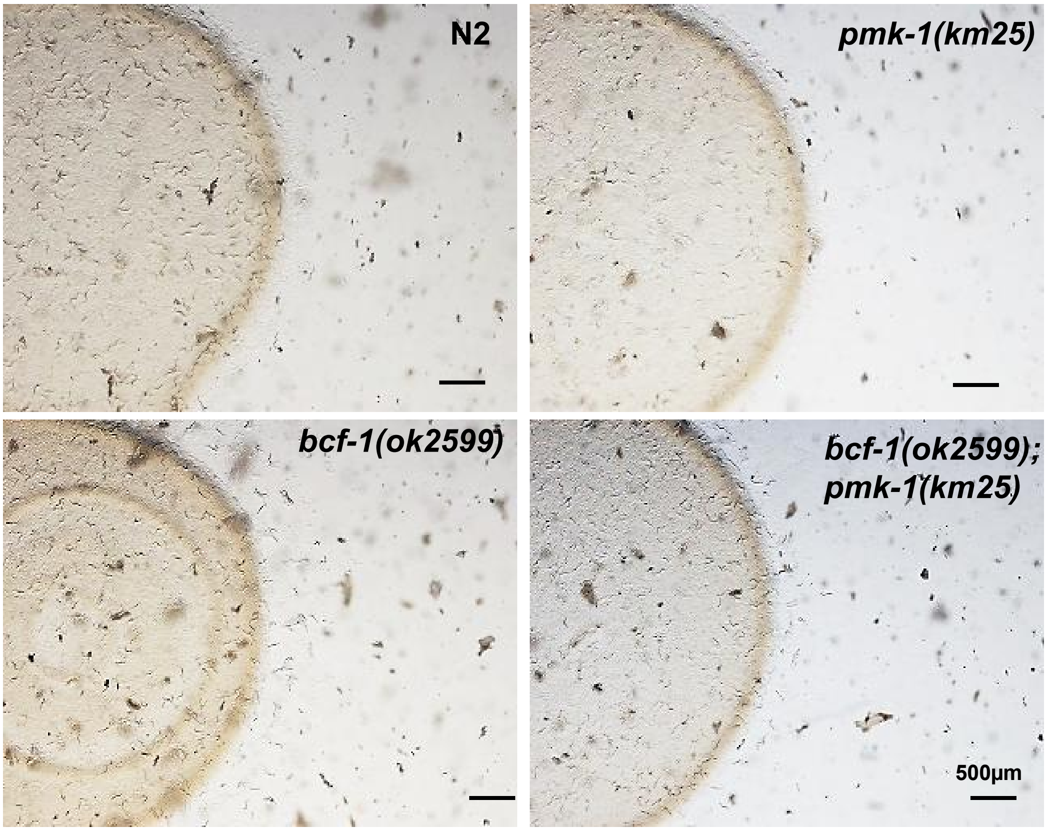

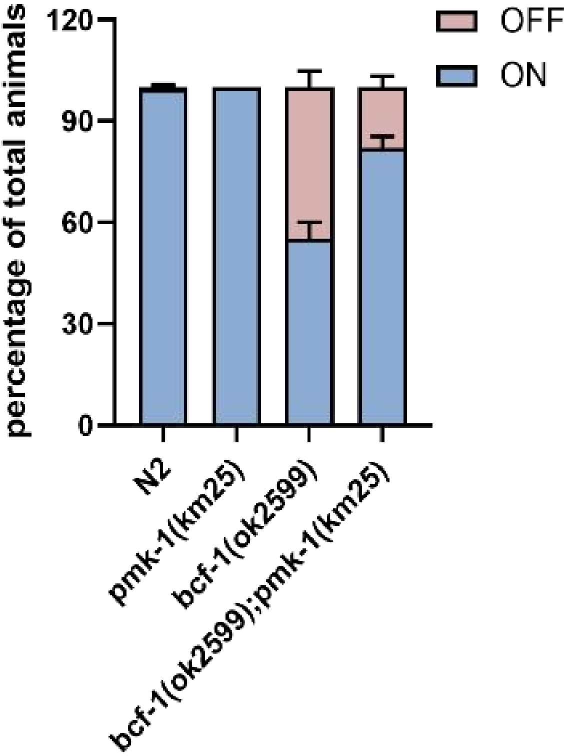

Supplementary Fig. 6: The food avoidance phenotype of *bcf-1(ok2599);pmk-1(km25)* double mutants**. Related to Fig. 6.**
Food avoidance is rescued in bcf-1 mutant animals after *pmk-1* mutation.
Source data are provided as a Source Data file.

Supplementary Fig. 7

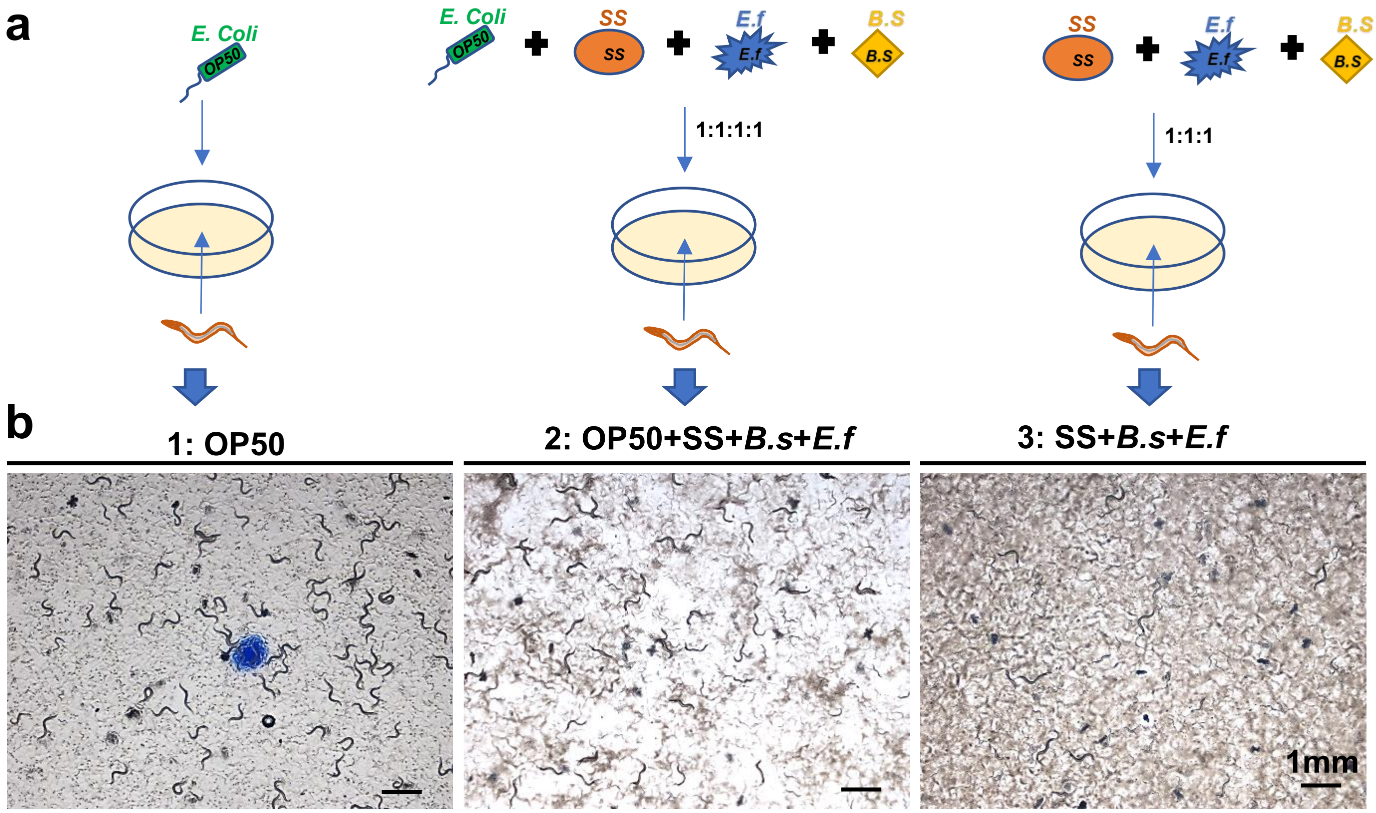

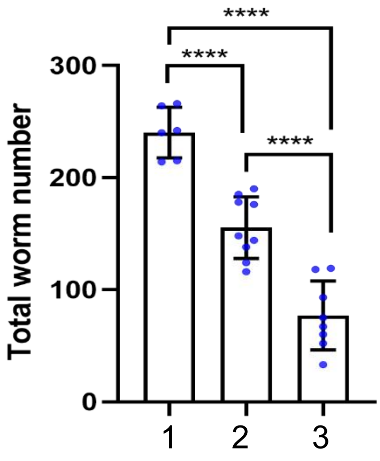

Supplementary Fig. 7: Diagram of adaptation analysis in animals feeding mixed bacteria to mimic nature environment. *E. coli, Staphylococcus saprophyticus (SS), Enterococcus faecalis (E.f)*, and *Bacillus subtilis (B.s) were mixed at indicated ratio to* mimic the natural food environment (**a**). Then a single wild-type N2 was seeded in the food. After 9 days culture, the number of animals were scored on the plate (**b**).**Related to Fig. 7.**
Data are represented as mean ± SD.
****p<0.0001, ns: no significant difference.
Source data are provided as a Source Data file.

Reference:

1 He, Y. et al. N-glycosylated intestinal protein BCF-1 shapes microbial colonization by binding bacteria via its fimbrial protein. *Cell Rep* 42, 111993 (2023).
