# Supplementary material for: Bacterial peptidoglycan acts as a digestive signal mediating host adaptation to diverse food resources in C. elegans
Source: Nat Commun. 2024 Apr 16;15:3286. doi: 10.1038/s41467-024-47530-y (PMC11021419; doi:10.1038/s41467-024-47530-y)
Supplement: Supplementary file 3 — Reporting Summary [file 41467_2024_47530_MOESM3_ESM.pdf]

nature portfolio

Corresponding author(s): Bin Qi

Last updated by author(s): Mar 24, 2024

Reporting Summary

Nature Portfolio wishes to improve the reproducibility of the work that we publish. This form provides structure for consistency and transparency in reporting. For further information on Nature Portfolio policies, see our Editorial Policies and the Editorial Policy Checklist.

Statistics

For all statistical analyses, confirm that the following items are present in the figure legend, table legend, main text, or Methods section.

n/a	Confirmed	
☐	☒	The exact sample size (n) for each experimental group/condition, given as a discrete number and unit of measurement
☐	☒	A statement on whether measurements were taken from distinct samples or whether the same sample was measured repeatedly
☐	☒	The statistical test(s) used AND whether they are one- or two-sided<br>*Only common tests should be described solely by name; describe more complex techniques in the Methods section.*
☐	☒	A description of all covariates tested
☐	☒	A description of any assumptions or corrections, such as tests of normality and adjustment for multiple comparisons
☐	☒	A full description of the statistical parameters including central tendency (e.g. means) or other basic estimates (e.g. regression coefficient) AND variation (e.g. standard deviation) or associated estimates of uncertainty (e.g. confidence intervals)
☐	☒	For null hypothesis testing, the test statistic (e.g. F, t, r) with confidence intervals, effect sizes, degrees of freedom and P value noted<br>*Give P values as exact values whenever suitable.*
☒	☐	For Bayesian analysis, information on the choice of priors and Markov chain Monte Carlo settings
☒	☐	For hierarchical and complex designs, identification of the appropriate level for tests and full reporting of outcomes
☒	☐	Estimates of effect sizes (e.g. Cohen's d, Pearson's r), indicating how they were calculated

Our web collection on statistics for biologists contains articles on many of the points above.

Software and code

Policy information about availability of computer code

Data collection	Analysis of fluorescence was performed with an Olympus BX53 microscope with a DP80 camera. Plate phenotypes were observed using an Olympus MVX10 dissecting microscope with a DP80 camera.Western blot images were observed using a MiniChemi 610.
Data analysis	Images:ImageJ Fiji(https://imagej.net/software/fiji/). Graphpad8.2.1(https://www.graphpad.com/updates/prism-821-release-notes)

For manuscripts utilizing custom algorithms or software that are central to the research but not yet described in published literature, software must be made available to editors and reviewers. We strongly encourage code deposition in a community repository (e.g. GitHub). See the Nature Portfolio guidelines for submitting code & software for further information.

Data

Policy information about availability of data

All manuscripts must include a data availability statement. This statement should provide the following information, where applicable:

- Accession codes, unique identifiers, or web links for publicly available datasets
- A description of any restrictions on data availability
- For clinical datasets or third party data, please ensure that the statement adheres to our policy

All data in main Manuscript and Supplementary information are listed in the Source data file. All reagents and strains generated by this study are available through request to the corresponding author with a completed Material Transfer Agreement. Source data are provided with this paper.

Research involving human participants, their data, or biological material

Policy information about studies with human participants or human data. See also policy information about sex, gender (identity/presentation), and sexual orientation and race, ethnicity and racism.

Reporting on sex and gender	Not applicable to this study
Reporting on race, ethnicity, or other socially relevant groupings	Not applicable to this study
Population characteristics	Not applicable to this study
Recruitment	Not applicable to this study
Ethics oversight	Not applicable to this study

Note that full information on the approval of the study protocol must also be provided in the manuscript.

Field-specific reporting

Please select the one below that is the best fit for your research. If you are not sure, read the appropriate sections before making your selection.

[x] Life sciences [ ] Behavioural & social sciences [ ] Ecological, evolutionary & environmental sciences

For a reference copy of the document with all sections, see nature.com/documents/nr-reporting-summary-flat.pdf

Life sciences study design

All studies must disclose on these points even when the disclosure is negative.

Sample size	Sample sizes for all analyses are determined following eatablished protocols and standard practice in C.elegans.Each experiment was set up as three groups of replicates, and the number of developmental statistics was more than 100 nematodes to exclude individual differences.For qPCR and Wetern blot, the number of worms used was determined according to our pre-tests.
Data exclusions	No data was excluded
Replication	In the main and supplementary figures,at least two biological replicates,each with at least three technical replicates,were included in the assays. In all large-scale screens,each strain was assayed twice. If the data from two replicates differ,a third assay was included.
Randomization	Synchronized worms were randomly distributed into experimental and control groups for each genotype
Blinding	Investigators were not blinded.Since the experimental data obtained for development required manipulation of live animals, blinding was not possible during data collection.

Reporting for specific materials, systems and methods

We require information from authors about some types of materials, experimental systems and methods used in many studies. Here, indicate whether each material, system or method listed is relevant to your study. If you are not sure if a list item applies to your research, read the appropriate section before selecting a response.

Materials & experimental systems

n/a	Involved in the study	
[ ]	[x]	Antibodies
[x]	[ ]	Eukaryotic cell lines
[x]	[ ]	Palaeontology and archaeology
[ ]	[x]	Animals and other organisms
[x]	[ ]	Clinical data
[x]	[ ]	Dual use research of concern
[x]	[ ]	Plants

Methods

n/a	Involved in the study	
[x]	[ ]	ChIP-seq
[x]	[ ]	Flow cytometry
[x]	[ ]	MRI-based neuroimaging

Antibodies

Antibodies used	Primary antibody:<br>anti-flag M2 monoclonal antibody(Sigma,F3165), diluted 1:3000 for immunoblotting;

Phospho-p38 MAPK (Thr180/Tyr182) (D3F9) XP® Rabbit mAb(Cell Signaling, 4511S), diluted 1:3000 for immunoblotting;
p38 MAPK Antibody(Cell Signaling,9212S), diluted 1:3000 for immunoblotting;
monoclonal anti-α-tubulin antibody(Sigma,T5168), diluted 1:3000 for immunoblotting;

Secondary antibody:
Goat anti-mouse antibody(Invitrogen, 626520), diluted 1:10000 for immunoblotting;
Goat anti-Rabbit IgG(H+L),HRP(ABclonal, A5014), diluted 1:10000 for immunoblotting.

Validation	Each antibody used in this study has been validated by the manufacturer and published elsewhere in the literature. Primary antibodies used were:<br>anti-flag M2(F3165)-sigma-Noted on the Sigma website to be used in both western blotting.And it has been previously used successfully in C.elegans,including He et al.,2023-https://doi.org/10.1016/j.celrep.2023.111993<br>Phospho-p38 MAPK(4511S)-Cell Signaling-Noted on the Sigma website to be used in both western blotting.And it has been previously used successfully in C.elegans,including Geng et al.,-2022-https://doi.org/10.1016/j.chom.2022.08.004<br>Phospho-MEK1/2 (Ser217/221) Antibody #9121-Noted on the Sigma website to be used in both western blotting.And it has been previously used successfully in C.elegans,including Geng et al.,-2022-https://doi.org/10.1016/j.chom.2022.08.004<br><br>Secondary antibody:<br>Goat anti-mouse antibody(Invitrogen, 626520),characterized specificity for mouse immunoglobulins and are useful in the detection<br>Goat anti-Rabbit IgG(H+L),HRP(ABclonal, A5014).Noted on the website to be used in both western blotting.

Animals and other research organisms

Policy information about studies involving animals; ARRIVE guidelines recommended for reporting animal research, and Sex and Gender in Research

Laboratory animals	Age-synchronized wild type and mutant C. elegans hermaphrodites were used in our tests. These include:N2,bcf-1(ok2599),bcf-1(ylf1),SJ4100 zcIs13 [hsp-6p::GFP + lin-15(+)],MGH171 alxIs9 [vha-6p::sid-1::SL2::GFP],SJ4197 zcIs39 [dve-1p::dve-1::GFP],syb4067 [F57F4.4::GFP::3XFLAG],bcf-1(ok2599); zcIs13,atfs-1(gk3094),atfs-1(et18).
Wild animals	Wild animals were not used in this study.
Reporting on sex	Hermaphrodite animals were used in all C. elegans tests.
Field-collected samples	Field-collected samples were not used in this study.
Ethics oversight	This study didn't involve materials or animal models that require ethical approval .

Note that full information on the approval of the study protocol must also be provided in the manuscript.

Plants

Seed stocks	NA
Novel plant genotypes	NA
Authentication	NA
