# Supplementary figures and images for: Bacterial peptidoglycan acts as a digestive signal mediating host adaptation to diverse food resources in C. elegans
Source: Nat Commun. 2024 Apr 16;15:3286. doi: 10.1038/s41467-024-47530-y (PMC11021419; doi:10.1038/s41467-024-47530-y)

Fig3C

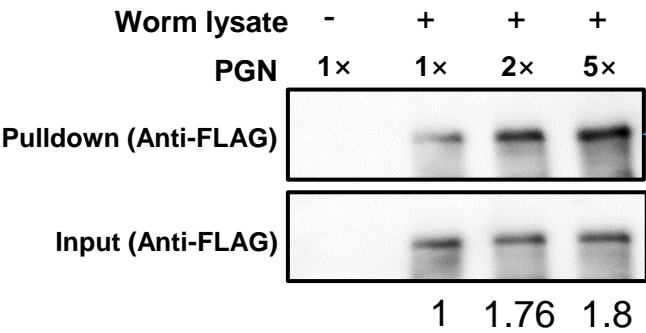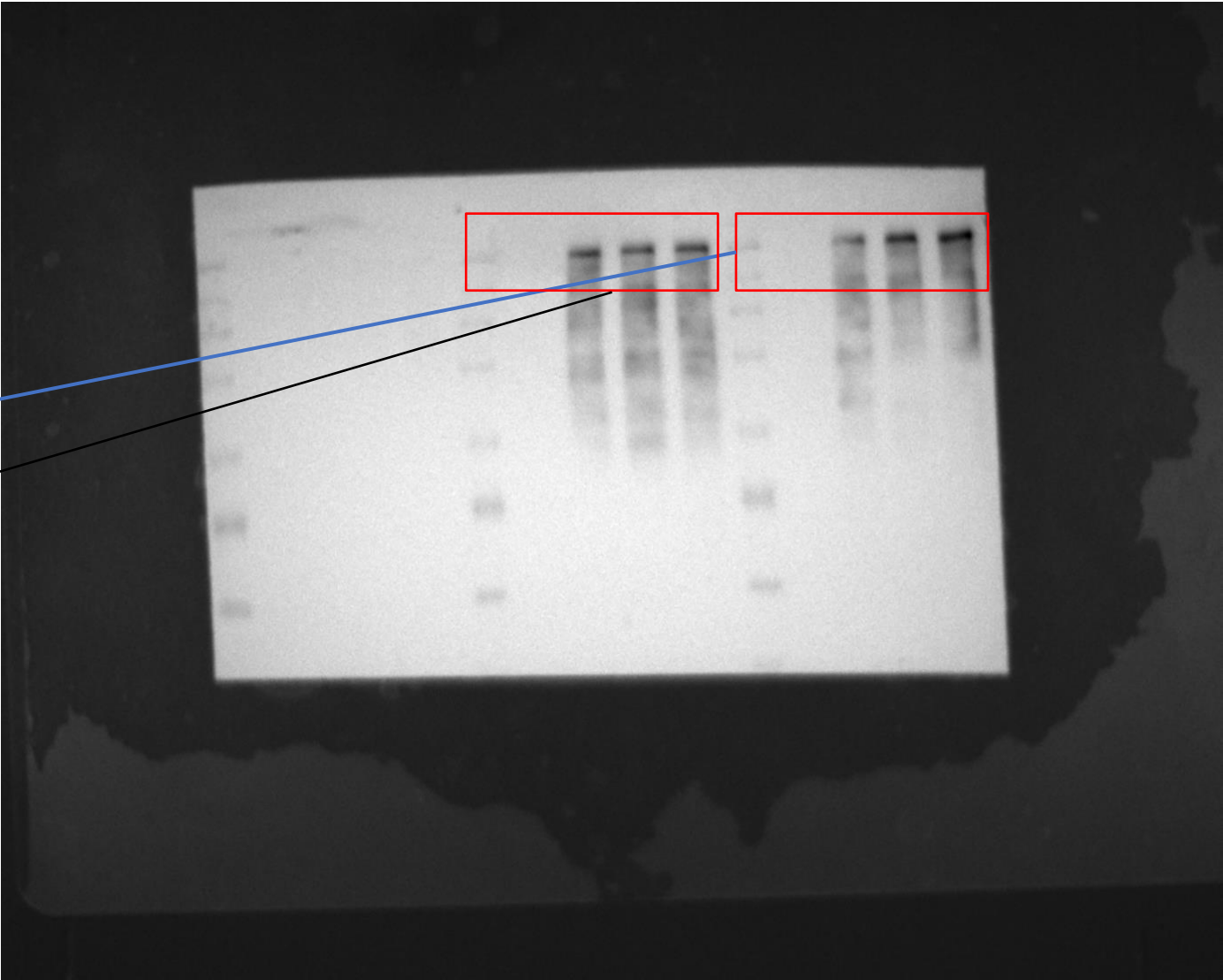

Fig3d

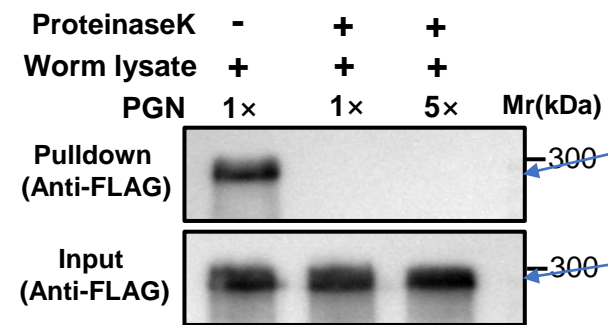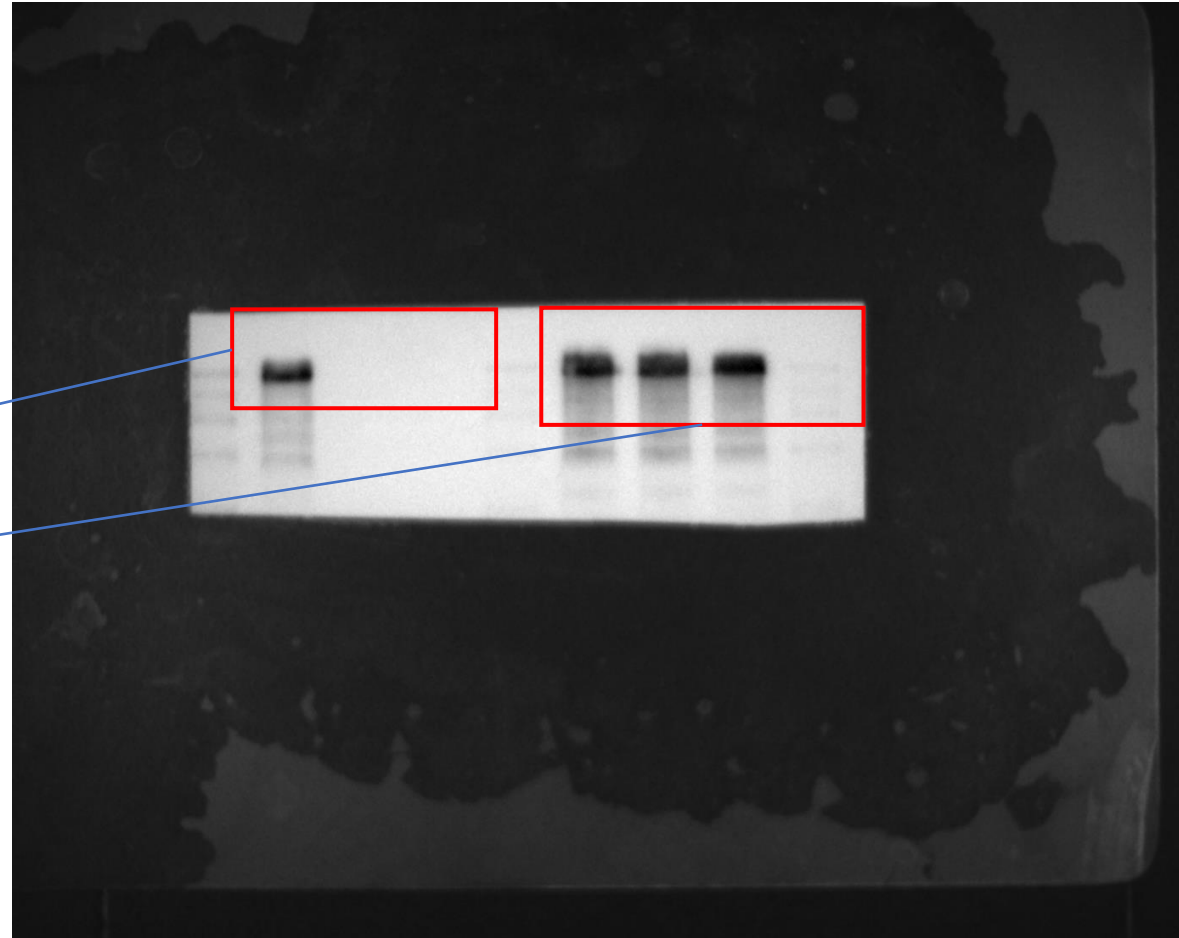

Fig3f

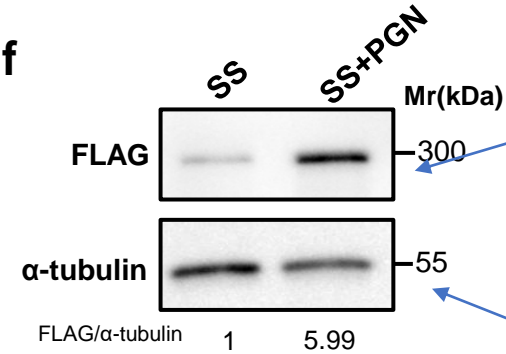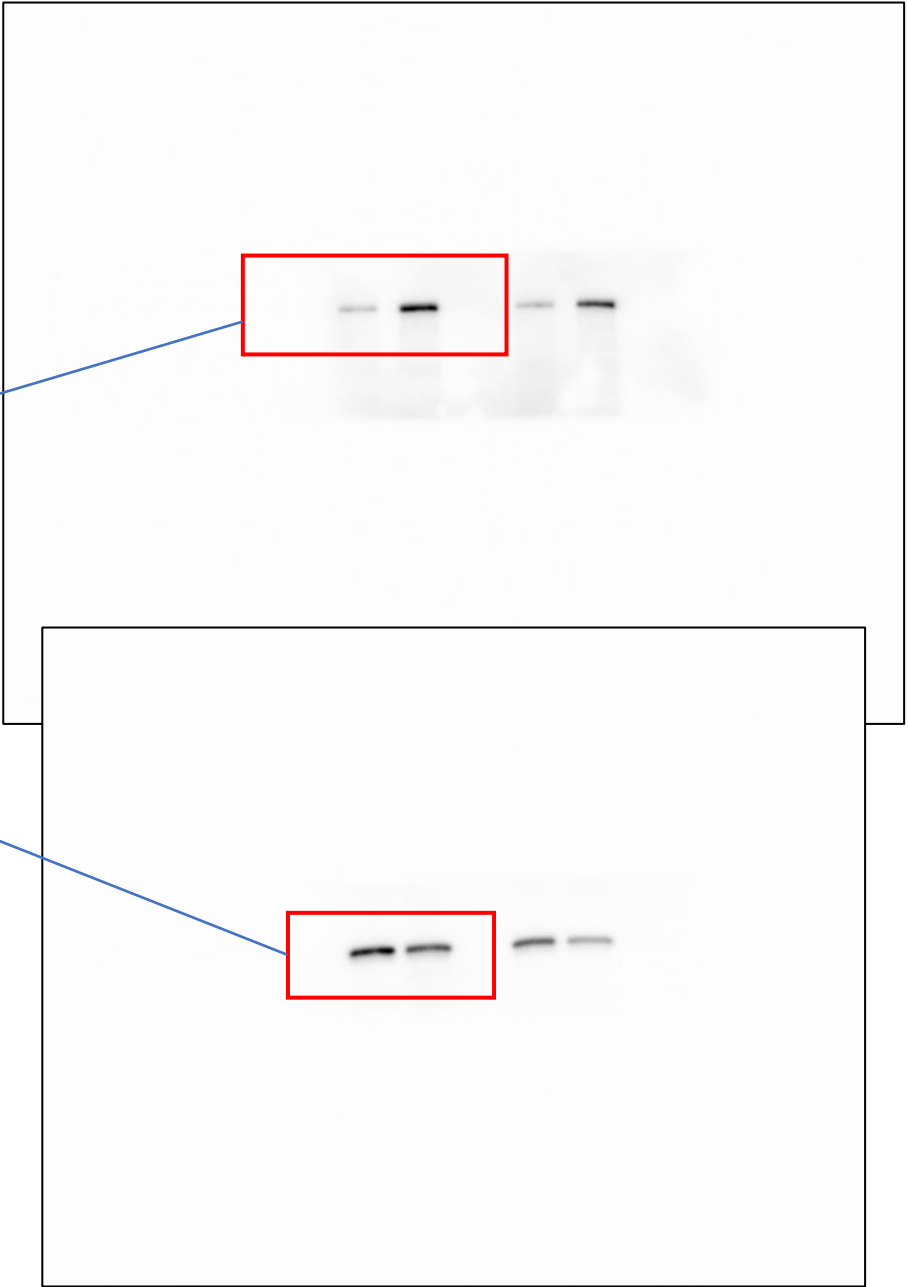

Fig6a

a

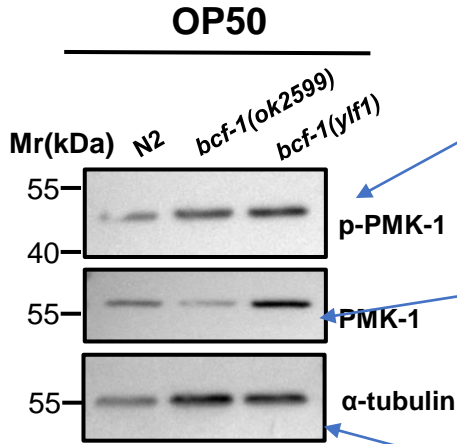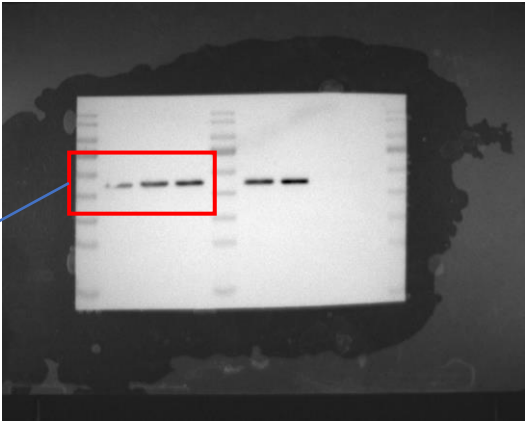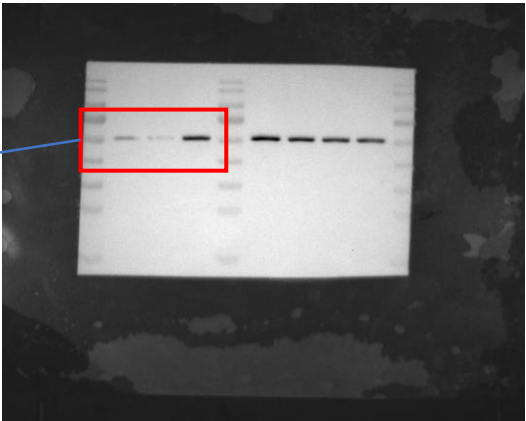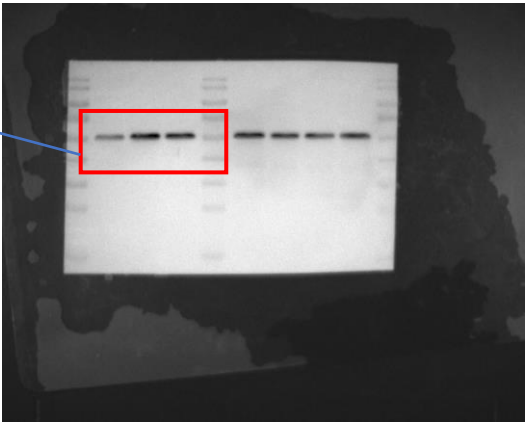

Fig6b

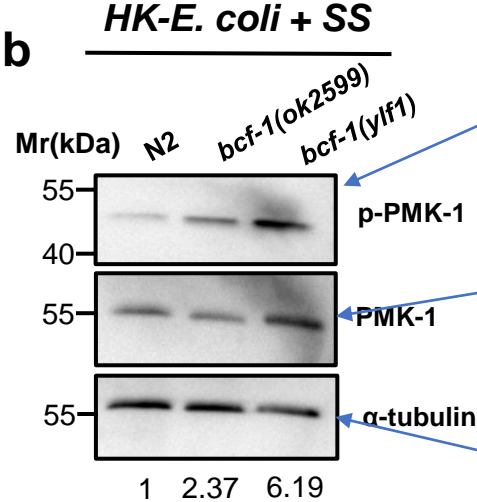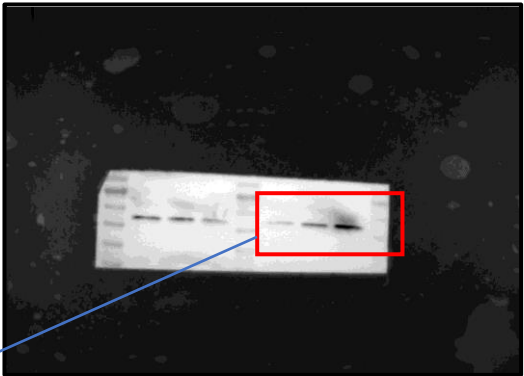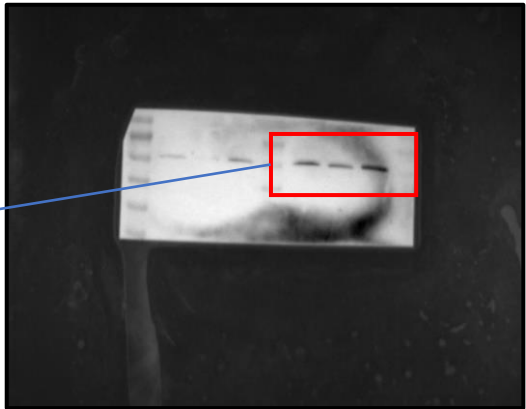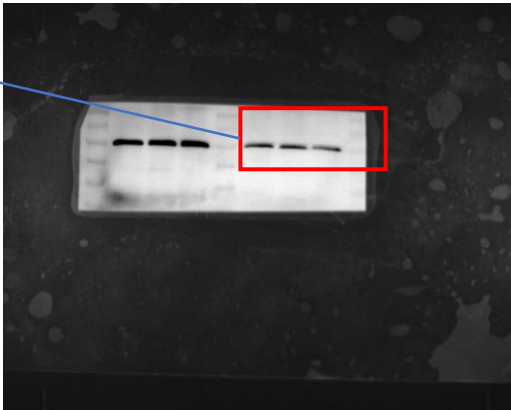

Fig6d

**d**

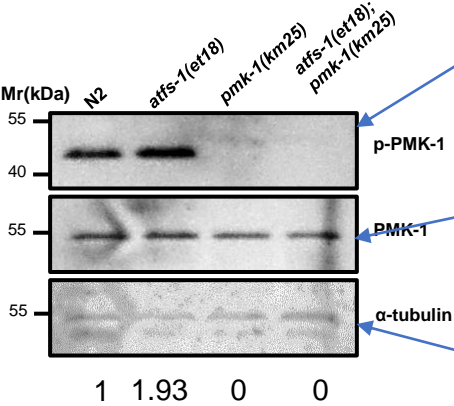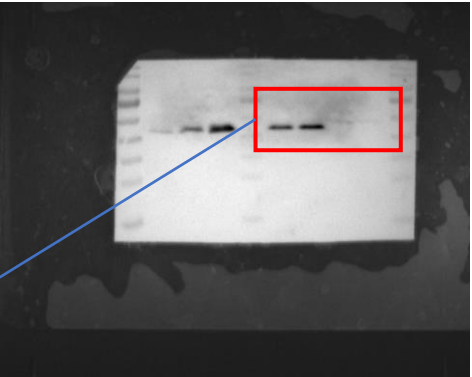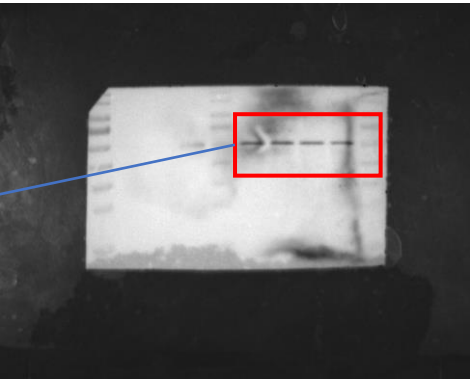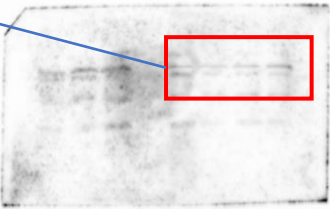

Supplement: Supplementary file 4 — Source Data [file 41467_2024_47530_MOESM4_ESM.zip › 6-source data-HFR/uncropped scans of western blot.pdf]
